# Understanding the Drivers of Food Waste in Malaysian Public Hospitals (MyHoFWa Study)

**DOI:** 10.21315/mjms-05-2025-379

**Published:** 2025-10-31

**Authors:** Nurul Alia Aqilah Samiun, Nurul Huda Razalli, Suzana Shahar, Zahara Abdul Manaf, Zurina Kefeli, Norshariza Jamhuri

**Affiliations:** 1 Dietetic Programme, Centre for Healthy Ageing and Wellness, Faculty of Health Sciences, Universiti Kebangsaan Malaysia, Kuala Lumpur, Malaysia; 2 School of Nutrition and Dietetics, Faculty of Health Sciences, Universiti Sultan Zainal Abidin, Gong Badak Campus, Kuala Nerus, Terengganu, Malaysia; 3 Faculty of Economics and Muamalat, Universiti Sains Islam Malaysia, Nilai, Negeri Sembilan, Malaysia; 4 Dietetic and Food Service Department, National Cancer Institute, Putrajaya, Malaysia

**Keywords:** food waste, hospital, healthcare, thematic analysis

## Abstract

**Background:**

Understanding the perspectives and experiences of hospital food service personnel in managing food waste may aid in determining the factors that influence food waste. However, such research is still scarce globally. This study aimed to determine the factors influencing food wastage in Malaysian public hospitals.

**Methods:**

This qualitative research was performed in four Malaysian public hospitals and used purposive sampling to select participants for one-to-one interviews because of their role as key informants.

**Results:**

Themes such as patient culture and background, the quality and variety of food choices, the complexity of the ordering system, staff competency and attitude, environmental factors, treatment procedures, and patient clinical condition were identified using thematic analysis.

**Conclusion:**

Rather than focusing solely on patient experiences, this study explores other factors influencing food wastage in Malaysian public hospitals based on the experiences of hospital food services staff in managing food wastage.

## Introduction

Food waste poses a serious problem, accounting for 27% to 32% of the food produced globally by weight or 24% by kilocalories (kcal) (1, 2). If global food waste is to be treated as its own nation, it would be the third largest emitter of greenhouse gases after China and the United States, occupy 30% of the world’s agricultural land area, and consume the equivalent water of Russia’s annual Volga River discharge (i.e., 250 km^3^) (3). Based on the average global food waste per capita per year, it could supply a person’s daily necessary intake (DRI) of 25 nutrients for 18 days, which includes 25% to 50% of a person’s DRI for vitamin C, K, zinc, copper, manganese, and selenium, depending on the types of food discarded (4). Estimates show that Malaysia’s food wastage reached 16,688 tonnes per day in 2017, an amount that increased by 15% to 20% during the holiday season (5).

Historically, hospital food waste has been a concerning issue in the healthcare industry, with food waste making up 50% of total waste by volume in some hospitals (6, 7). Due to their size, complexity, and the nature of their operations, hospitals can generate substantial food waste. This waste accumulates at various stages, including food preparation, tray assembly, patient consumption, and kitchen cleanup (8). The food waste includes uneaten meals, spoiled or expired food, and excess food prepared but not served, all of which contribute to overall food waste (9).

Majority of the hospitals nowadays are striving to improve food quality and delivery systems to achieve patient satisfaction and acceptance while also reducing the cost of food wastage (10). The two most common plating systems in hospital food service are centralised and decentralised systems. The centralised system incorporates preparing and serving meals on plates in the kitchen, while the decentralised system delivers food to wards using portable warmers or trolleys (11). In Malaysia, hospital food services are generally run either by in-house management or outsourced to a third party. In-house management refers to a catering system in which the hospital oversees manpower, facilities, and operations. Conversely, outsourced management allows a private company to bring in its own manpower and facilities to operate within the hospital vicinity, often involving a large staff dedicated to delivering and serving food in the wards and catering to a large number of patients (12).

Previous studies have documented that high food waste rates in hospitals were attributed to several variables, including patient clinical conditions, environmental problems, food quality, and menu issues (13). According to published research, hospital plate waste exceeding 30% is deemed high (14). Compared with other food service industries, hospital facilities have a higher food waste rate, ranging from 6% to 65% (13). A previous Malaysian study found that the average plate waste among inpatients was about 50% (15), consistent with recent findings (7, 16, 17). A previous local research on hospitalised geriatric patients in Malaysia revealed varying food waste rates across courses, ranging from 20% to 90%, with protein dishes resulting in the highest waste (18). Another recent study conducted in Malaysian public hospitals found that the largest plate waste was protein and vegetable (86.7%), while the lowest plate waste was fruit (73.4%) (19).

Notably, hospital food waste has always been associated with plate waste and is based on patient satisfaction (8, 16). Research on hospital food waste, especially in Malaysia, remains limited. The majority of studies focus on quantitative plate waste data rather than the factors behind the issues of high food waste in Malaysian public hospitals. Thus, this study aimed to determine the factors influencing food wastage in Malaysian public hospitals.

## Methods

### Study Design

An inductive technique was used for this qualitative study, which focuses on a smaller, open-ended, and exploratory sample, allows the emergence of different explanations for a specific situation. To achieve the main objective of identifying the factors influencing food wastage in Malaysian public hospitals, in-depth semi-structured interviews were conducted.

### Study Setting

**Figure 1:**
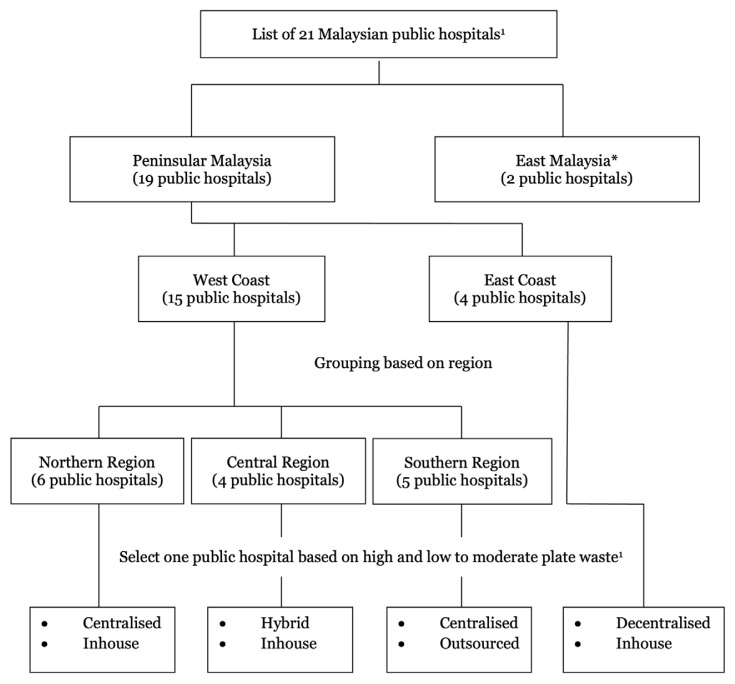
Study location *Hospitals from East Malaysia had to be eliminated due to restrictions during the Movement Control Order of the COVID-19 outbreak; ^1^ Source from Dietitian’s Quality and Research Bureau through personal communication with a Ministry of Health senior dietitian

Data collection took place between 2020 and 2021 in four public hospitals in Peninsular Malaysia. Data collection was prolonged due to the COVID-19 Movement Control Order. As illustrated in Figure 1, hospitals were selected from a shortlist of 21 public hospitals representing various state and specialist government facilities, categorised by their food service systems (centralised, decentralised, in-house, and outsourced). These 21 public hospitals were then categorised by region, including the northern, central, southern, and west coast regions. One public hospital was selected to represent each region based on its high, low or medium plate waste. The list of 21 Malaysian public hospitals and unpublished data on the plate waste study were obtained from the Dietitian’s Quality and Research Bureau through personal communication with a Ministry of Health senior dietitian.

Data saturation was the guiding principle for determining the sample size in this qualitative study. Data collection ceased when no new information emerged from the interviews. The number of staff involved was estimated according to the availability of staff in the hospital. The food service personnel were categorised into two groups: management staff, who establish the Standard Operating Procedures (SOPs) for service systems, and operational staff, who execute these SOPs. Participants were selected using purposive sampling due to their roles as key informants.

The following staff groups were identified as having relevant knowledge and information regarding the issue of hospital food waste:

- Catering officers, foodservice manager: Management staff, responsible for the operational aspects of food services;
- Dietitians: Management staff, responsible for providing dietary advice and monitoring the diet given to patients;
- Nurses: Management staff, responsible for taking patients’ food orders;
- Cooks: Operational staff, responsible for cooking and portioning food to the wards;
- Foodservice staff: Operational staff, responsible for portioning and delivering food to patients

The interview protocols were developed using the available literature and data from numerous discussions with a dietitian from the Malaysian Ministry of Health. The protocols covered key areas, including participants’ roles in food waste, their perceptions of hospital food waste, the factors influencing it, and methods for reducing it. The protocols delve into thoughts on food waste (including challenges and opportunities), decision-making processes, staff training for waste management, and recent changes to waste management procedures. The interview protocols for management and operational staff were quite similar, with the primary difference being questions on the administration of the food service system in Malaysian public hospitals. The interview questions are included in Table 1. Prior to the data collection, the interview process was tested by interviewing two dietitians to identify any ambiguities. Before the interview, each participant received study information and consent papers.

### Recruitment of Respondents

The majority of respondents were located and enlisted with assistance from dietitians, catering officers, or ward sisters (nurses). Eligibility criteria included fluent communication in Malay or English and a minimum of two years’ work experience in hospital food service. Researchers eventually contacted eligible individuals and conducted interviews if they met the inclusion/exclusion criteria and agreed to participate. The interviews took place in the staff area during the employees’ leisure time and lasted roughly 20 to 30 minutes each. Sessions ended when all relevant topics had been addressed and data saturation (when all participants provided the same answers) was achieved. Before the data was analysed, interviews were digitally recorded, verbatim transcribed, and then translated (from Malay to English).

### Data Analysis

The data were organised, explored, integrated, and eventually interpreted using ATLAS.ti software. ATLAS.ti helped with the thematic review process by hyperlinking the initial coding into “themes.” Prior to further analysis and reduction to emergent themes, results were first categorised according to the research questions.

## Results

### Profile of Participants

The profiles of participants interviewed are presented in Table 2. The number of participants in each staff group was based on the availability of hospital employees during data collection. A total of 16 food service personnel from four different Malaysian public hospitals were interviewed in this study. A total of five catering officers, four dietitians, three cooks, two food service personnel, one food service manager, and one nurse were interviewed. The majority of participants were females (62.5%), while males were 37.5%. Their average age was 40.6 (11.2) years, with an average of 14.1 (10.0) years of working experience. Most of the participants had tertiary education, while others had secondary education. About 68.7% of participants worked in an in-house setting, and 31.3% worked in an outsourced setting. The majority of participants work in centralised plating kitchens (56.2%), while others work in hybrid (37.5%) or decentralised plating kitchens (6.3%). Overall, the median interview duration was 15 minutes 24 seconds.

### Factors Leading to Food Waste in Malaysian Public Hospitals

**Figure 2:**
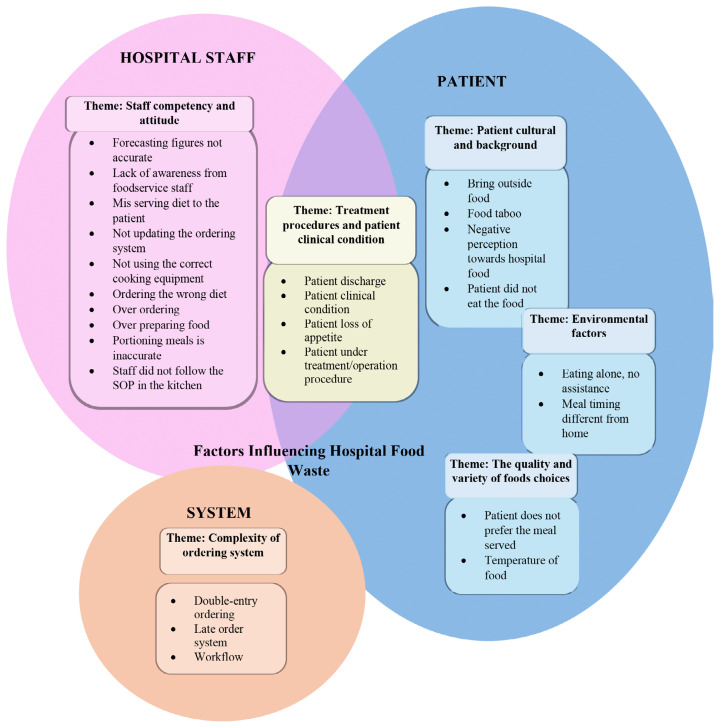
Themes and sub-themes

The results were classified into six emerging themes (Figure 2):

- Patient culture and background;
- The quality and variety of food choices;
- Complexity of the ordering system;
- Staff competency and attitude;
- Environmental factors; and
- Treatment procedures and patient clinical condition.

#### Patient Cultural and Background

The kitchen staff and higher management agreed that patient personal preferences are a primary reason for hospital meal waste. This challenge is amplified in Malaysia by its multiracial population and varied cultural palates.

Even if you serve a full diet (normal diet) to these people (patients), they will still complain due to their background. For example, when we serve *nasi tomato* (tomato rice), the Indians rejected it because they do not eat *nasi tomato*. (Dietitian 1, in-house hospital, hybrid catering)

The food service personnel also informed that many patients complained that the hospital food was tasteless.

The patient doesn’t want to eat because they prefer outside foods. They said that the hospital food does not add any salt and is tasteless. For example, we order a low salt diet for hypertension patients, but they do not want to eat because it is not up to their preference. (Nurse 1, in-house hospital, hybrid catering)

It is interesting to note that the food service personnel mentioned that certain patients have negative perspectives on hospital food, alongside some food taboos, particularly for post-operation meals.

Besides that, food wastage also occurs when families bring outside food to the patients. Both kitchen staff and higher management mentioned that when this occurred, hospital meals would remain untouched. Even without family-provided meals, patients could still order food delivery, which is very popular nowadays.

They (patients) said that they have their own preferences and want their children or relatives to bring food to them. That is what they (patients) prefer. (Cook 1, in-house hospital, hybrid catering)

We already did the best (preparing food) and it was delicious, but they (patients) still do not want to eat. Maybe because they ate outside food. (Cook 3, in-house hospital, centralised catering)

#### The Quality and Variety of Food Choices

The kitchen staff and higher management reported frequent patient complaints about the quality and limited variety of food choices in hospital meals. Higher management further claimed that vegetables were the most wasted food item.

The patients seem not to like the vegetables served to them. But it still depends on the types of vegetables. Or maybe they do not like the way we prepared the meals. (Catering officer 4, outsourced hospital, centralised catering)

Usually, cabbage get wasted because they (patients) complain that cabbage makes them feel nauseous. (Catering officer 2, in-house hospital, hybrid catering)

Beyond complaints about tasteless therapeutic diet, higher management also believed food temperature played an important role. Based on their observations, they could not maintain the food’s temperature due to limited time and kitchen equipment.

I think that wastage happens a lot on the therapeutic diet because of the taste and temperature. (Catering officer 1, in-house hospital, hybrid catering)

Maybe the food was wasted because they (patients) do not like the way we served the meals. It is no excuse that the therapeutic diet, especially low salt diet, is tasteless. (Catering officer 4, outsourced hospital, centralised catering)

One higher management disagreed when the nurse complained that the food was wasted because the menu cooked and served was not delicious.

When we pointed out that there was a problem of double entry in the menu order, they started to blame us, saying that our food was not delicious. We then lost for words. They said that our menu is not delicious. (Catering officer 3, in-house hospital, centralised catering)

#### Complexity of the Ordering System

There is no denying that the food service staff struggled with the complexity of the ordering systems. Every hospital has its own system for ordering patient meals. While many hospitals have transitioned to computerised systems, some still rely on manual systems (or are in the process of transition). Regardless of the system, these methods still have a potential for error.

What has been ordered (diet order) is not the same as in the system. They (the nurse) seem confused with the ordering system. (Catering officer 1, in-house hospital, hybrid catering)

Let me show you (show ordering sheet). This is a double entry. Under one patient, there are two orders ybeen done. (Catering officer 3, in-house hospital, centralised catering)

Recently, we made some changes to the ordering sheet for improvement. So, these changes are quite confusing for the staff, and it takes some time for them to understand to use it. (Catering officer 1, in-house hospital, hybrid catering)

We have reminded them (nurses) to make orders in the computerised ordering system, but the system is not helpful (not friendly user). So, they (nurses) make orders manually. If they do it manually, they can just easily manipulate the number (diet order). (Catering officer 1, in-house hospital, hybrid catering)

#### Staff Competency and Attitude

Higher management had several comments regarding the staff competency. Although annual refresher courses and training are being conducted to reinforce staff responsibilities, issues regarding staff competency and attitude still arises.

Food wastage might happen because of an error in counting. For example, we ask the staff to take out 400 pieces of chicken for a normal diet and 200 pieces of chicken for a therapeutic diet. While counting, they are chatting with each other (not focusing on their task). We already reminded them, but still happened. Sometimes they also count it backwards. (Catering officer 3, in-house hospital, centralised catering)

Sometimes the portion that we give to the patient is big. The measurement used in the ward is also not accurate. Not using weight, just estimation. So food wastage could happen there. (Cook 1, in-house hospital, hybrid catering)

We have tagged all of the patients’ plates with the right order, but when they arrive at the ward, the staff there collect all the tagging away and serve food to the patients according to their ways. Means that some patients might not get the right order. (Cook 3, in-house hospital, centralised catering)

I can say that they (kitchen/ward staff) are lacking awareness in handling food wastage. They do not think that it is important to give the right diet to the right patients. The same goes during the preparation. They did not follow the SOP to cut vegetables or chicken, or use the right equipment to cook. (Catering officer 1, in-house hospital, hybrid catering)

Our staff comes from various backgrounds, so their attitudes are quite challenging to tackle. Their knowledge of food service is also low. They just want to do the job for the sake of money. (Dietitian 4, outsourced hospital, centralised catering)

The food service personnel also said that nurses were never informed when any patients were discharged or could not have meals due to medical treatments.

Nurse did not call us (update) if there is a discharge. But only call if the food was not enough. (Dietitian 4, outsourced hospital, centralised catering)

They (the nurse) did not update the ordering system when the patient was discharged. This leads to wastage. (Catering officer 2, in-house hospital, hybrid catering)

However, the nurse responded that they were too busy with their tasks and did not have time to update the diet order.

The situation in the ward is busy. We do not have the time to call the kitchen, “Please do not give this patient food because he just undergoing an operation.” (Nurse 1, in-house hospital, hybrid catering)

#### Treatment Procedures and Patient Clinical Condition

Another reason that makes food wastage difficult to control in hospitals was the unpredictable nature of treatment procedures and patient conditions. The nurse described that even when they placed correct meal orders, patients sometimes had to be transferred for operations or medical procedures, leaving their beds (and meals) untouched.

Patients have lots of procedures to do in hospital. Sometimes they need to go for an operation, ultrasound, and others. So, if they come back late, we tend not to serve them meals just to avoid food poisoning. So, this plate we are thrown away. (Nurse 1, in-house hospital, hybrid catering)

Sometimes the food is wasted because the patient has already been discharged. (Dietitian 4, outsourced hospital, centralised catering)

We usually did a forecasting for tomorrow’s breakfast based on the number of patients at yesterday’s dinner. There might be some patients who are discharged (we are not aware of this). So, of course, there will be leftover food. (Dietitian 2, in-house hospital, centralised catering)

#### Environmental Factors

Environmental factors such as hospital odours, feelings of loneliness, and hospital mealtimes can also play roles in hospital food wastage.

Some patients said that hospital food smells like medicine. I know that the hospital always disinfects the floor. Maybe the smell is absorbed into the food even though it has already been covered. Patients tend to be quite sensitive. (Dietitian 1, in-house hospital, hybrid catering)

Usually they (patients) eat with family during mealtimes, but here must eat alone. Furthermore, the mealtime is not their usual mealtime at home. (Dietitian 1, in-house hospital, hybrid catering)

## Discussion

This study investigated hospital food service staff’s perceptions, beliefs, and experiences with food waste. The factors contributing to hospital food waste were investigated based on the daily experiences of food service employees, from preparation to serving meals. Both management and operational staff were interviewed to determine the underlying causes of excessive hospital food waste. The participants engaged with topics on current challenges of food waste in hospitals and potential future solutions, and they provided insights they could elaborate on. This aligns with the third target of SDG 12, “ensure sustainable consumption and production patterns,” which directs all countries to reduce food losses across production and supply chains (including post-harvest losses) and to halve global per capita food waste at the retail and consumer level by 2030 (20).

The present study shows the significant influence of patients on hospital food waste, given their role as primary consumers in this setting. Patients were central to themes such as patient culture and background, the quality and variety of food choices, as well as environmental factors. This aligns with the existing literature, which includes numerous studies exploring patients’ perspectives on hospital food waste (8, 16, 21, 22). The present study found that the food service personnel acknowledged patients’ preferences and complaints about hospital food. Malaysia’s multicultural demographics present a unique challenge for dietitians in creating menus that cater to diverse cultural palates (23). When hospital menus do not align with specific food preferences or dietary norms from various cultural backgrounds, patients may reject certain meals, resulting in uneaten food and waste.

Every culture likely has its share of food taboos, which are traditional or cultural standards that restrict or discourage the consumption of specific foods (24). For example, pregnant women may avoid foods deemed hazardous to the mother or child due to cultural, traditional, or religious beliefs (25, 26). Thus, food service staff have little choice but to avoid specific types of food that the patients would refuse. This can be accomplished by surveying which foods produce the most waste and understanding the reasons behind it.

Negative perceptions of hospital food can arise from the ward environment itself. A previous study found that the surroundings influence the entire eating experience (27). The constant presence of various medications, prescribed hourly and daily, might alter patients’ taste perception due to their odours. Furthermore, food waste often occurs when patients receive outside food from family or friends. Patients tend to eat the outside food while still receiving hospital meals, leading to the hospital meals being wasted.

Hospital portion sizes often do not align with what patients typically consume. The amount of plate waste generated is directly related to the meal portion size (28). If portions are too large or too small, patients may discard leftovers. Past literature frequently highlights food quality and variety as key influencers of hospital food waste (8, 13, 29). The current study found that patients complained about the variety and ways of cooking vegetables, finding them unpalatable. Food temperature also impacted their appetite. Hospitals often struggle to meet patient expectations regarding food quality. Limited menus and a lack of variety in meal options can lead to monotonous food choices, causing long-stay patients to tire of repetitive meals. To combat this, hospitals should focus on offering diverse menus with a range of choices that cater to different dietary needs, cultural preferences, and tastes. Emphasising the quality of ingredients, food preparation techniques, and meal presentation can significantly impact patients’ willingness to eat hospital food.

Nurses in the current study reported being too busy to inform the kitchen of patients’ meal orders, making it challenging for kitchen staff to plan ahead. Most uneaten meals were due to patients’ medical conditions during treatment or at discharge. Meal forecasts were often inaccurate because patient circumstances— such as discharge, hospitalisation, relapse, and changes in appetite—could shift unexpectedly within three days (30). This forces kitchen staff to order for the maximum ward capacity, often exceeding actual demands and contributing to food waste (13, 31).

Several past literature highlight that staff competency and attitude are crucial for efficiently managing hospital food services and minimising food waste (29, 32–34). Incompetent or inadequately trained kitchen staff can lead to improper food handling, preparation, or storage, resulting in food spoilage and waste. Similarly, staff responsible for serving meals may lack the necessary training or understanding for proper portion control. Serving overly large portions that exceed patient needs can result in uneaten food and waste.

Our recent study found that most hospitals grappled with issues such as double ordering, late order systems, and constantly changing workflows. These technical glitches in the ordering process may lead to inaccurate meal orders. Previous studies have shown the benefits of technological upgrades; for instance, switching from a bedside spoken meal ordering system to an electronic system in a 126-bed public acute care adult hospital showed positive outcomes across nutritional intake, plate waste, staff and patient satisfaction, and patient food costs (35). Therefore, proper tracking and monitoring of food orders are essential to prevent overordering or underordering.

### Strengths and Limitations

The use of semi-structured interviews in this study allowed for a deep exploration of participants’ experiences, perceptions, and meanings of hospital food waste. However, since it is a non-random sample, the findings may not be generalisable to broader populations and may be limited to public hospital settings. For future studies, comparing different foodservice used in public and private hospitals may be beneficial.

## Conclusion

In this study, we conducted semi-structured interviews with food service personnel to explore their perspectives on the factors contributing to hospital food waste. We identified six key factors: patient culture and background; the quality and variety of food choices; the complexity of the ordering system; staff competency and attitude; environmental factors; and patient treatment procedures and clinical conditions. These factors were categorised into three main groups: hospital staff, patients, and the system. While many of these factors are patient-related, the roles of hospital staff and systemic issues are also significant. This study highlights that food waste in Malaysian public hospitals is influenced by a combination of factors, not solely by patient experiences. For future research aimed at reducing hospital food waste, it is crucial to consider the roles of staff and systemic factors, recognising that the issue of food waste is multifaceted and involves multiple stakeholders, not just patients, which will be key to developing more effective strategies.

## Figures and Tables

**Table 1:** Line of questioning for hospital food waste semi-structured interviews

No.	Line of questioning for hospital food waste
1	In your opinion, is there any food waste in the hospital? If yes, at what stage does it happen? If not, why do you think no food was wasted in the hospital?
2	As a (state their occupation), what is your perception when you encounter food wastage in the hospital?
3	What do you think about the staff’s attitude towards hospital food wastage? Is there any issue with overordering diet?
4	Why do you think hospital food waste occurs?
5	What is your role (state their occupation) in reducing hospital food waste?
6	Is there any guideline given to staff to reduce food waste? And is it efficient?
7	Did the management have set out strategies for reducing the hospital food waste? Please mention all the strategies.
8	Are there any challenges in reducing hospital food waste?
9	Are you aware of what is happening to the food wasted? Is there any action taken in handling the food waste?

**Table 2:** Profile of participants

Variables	*n* (%)
**Gender**	
Female	10 (62.5)
Male	6 (37.5)
**Age**	40.6 (11.2)*
**Education**	
Secondary	4 (25)
Tertiary	12 (75)
**Occupation**	
Cook	3 (18.7)
Foodservice staff	2 (12.5)
Catering officer	5 (31.2)
Foodservice manager	1 (6.3)
Dietitian	4 (25)
Nurse	1 (6.3)
**No years of working**	14.1 (10.0)*
**Type of food service**	
In-house	11 (68.7)
Outsourced	5 (31.3)
**Type of food preparation**	
Hybrid	6 (37.5)
Centralised	9 (56.2)
Decentralised	1 (6.3)

* values are in mean (SD)
